# RepBox: a toolbox for the identification of repetitive elements

**DOI:** 10.1186/s12859-023-05419-5

**Published:** 2023-08-22

**Authors:** Shelvasha Burkes-Patton, Elizabeth A. Cooper, Jessica Schlueter

**Affiliations:** 1 Department of Bioinformatics and Genomics, University of North Carolina at Charlotte, Charlotte, NC 28223 USA; 2 North Carolina Research Campus, Kannapolis, NC 28081 USA

**Keywords:** Transposable elements, Genomics, Detection software, Bioinformatics pipeline

## Abstract

**Background:**

Transposable elements (TEs) are short, mobile DNA elements that are known to play important roles in the genomes of many eukaryotic species. The identification and categorization of these elements is a critical task for many genomic studies, and the continued increase in the number of de novo assembled genomes demands new tools to improve the efficiency of this process. For this reason, we developed RepBox, a suite of Python scripts that combine several pre-existing family-specific TE detection methods into a single user-friendly pipeline.

**Results:**

Based on comparisons of RepBox with the standard TE detection software RepeatModeler, we find that RepBox consistently classifies more elements and is also able to identify a more diverse array of TE families than the existing methods in plant genomes.

**Conclusions:**

The performance of RepBox on two different plant genomes indicates that our toolbox represents a significant improvement over existing TE detection methods, and should facilitate future TE annotation efforts in additional species.

**Supplementary Information:**

The online version contains supplementary material available at 10.1186/s12859-023-05419-5.

## Background

Transposable elements (TEs) are defined as sequences of DNA capable of changing location within a given genome. Due to this mobility, researchers often refer to these sequences as “jumping genes” [1], with some being implicated in interference of gene function when inserting into coding regions. There are currently many different known classes of TEs [2], each with a slightly different sequence structure or mechanism of mobility, and numerous studies over the past few years have demonstrated the impact that different TEs can have on the evolution and expression of genes in eukaryotic species [3–5]. Due to their potential to disrupt or alter gene functions, the detection and classification of these elements in the genome is essential for researchers looking to better understand their characteristics and the roles that they play.

The importance of TEs has led to the creation of many tools and software packages which use both de novo and homologous methods to efficiently analyze genomic data for TEs [6, 7], but many of these programs forego the implementation of family-specific structural information to aid in identification, resulting in large numbers of elements classified as “unknown” [8]. A possible solution to this problem is to run multiple different family-specific programs independently, however this can require multiple software installations and customized data formatting for each tool, which can quickly become difficult or even impossible for users with limited computational experience [9].

To address these issues in TE identification, we developed RepBox, a Python suite of scripts that incorporate family-specific TE detection software. RepBox was designed as a user-friendly easily installable suite that expands the methods of identification to provide a simple and straight forward means of incorporating several different approaches to TE annotation with the underlying goal of reducing the unclassified/unknown element classes. We then compared the results of our RepBox pipeline and the existing RepeatModeler software on two plant genomes with well-curated TE databases. Our pipeline represents the following contributions to the field of transposable elements:

- RepBox supports the output generated by other TE identification software and is easily implemented without large disruption of established pipelines.
- RepBox is capable of identifying more diverse families of repetitive elements in the species we compared than RepeatModeler alone.
- RepBox re-classifies transposable element families previously identified as “unknown” by integrating multiple family-specific methods into one analysis.

## Implementation

### Pipeline overview

**Fig. 1:**
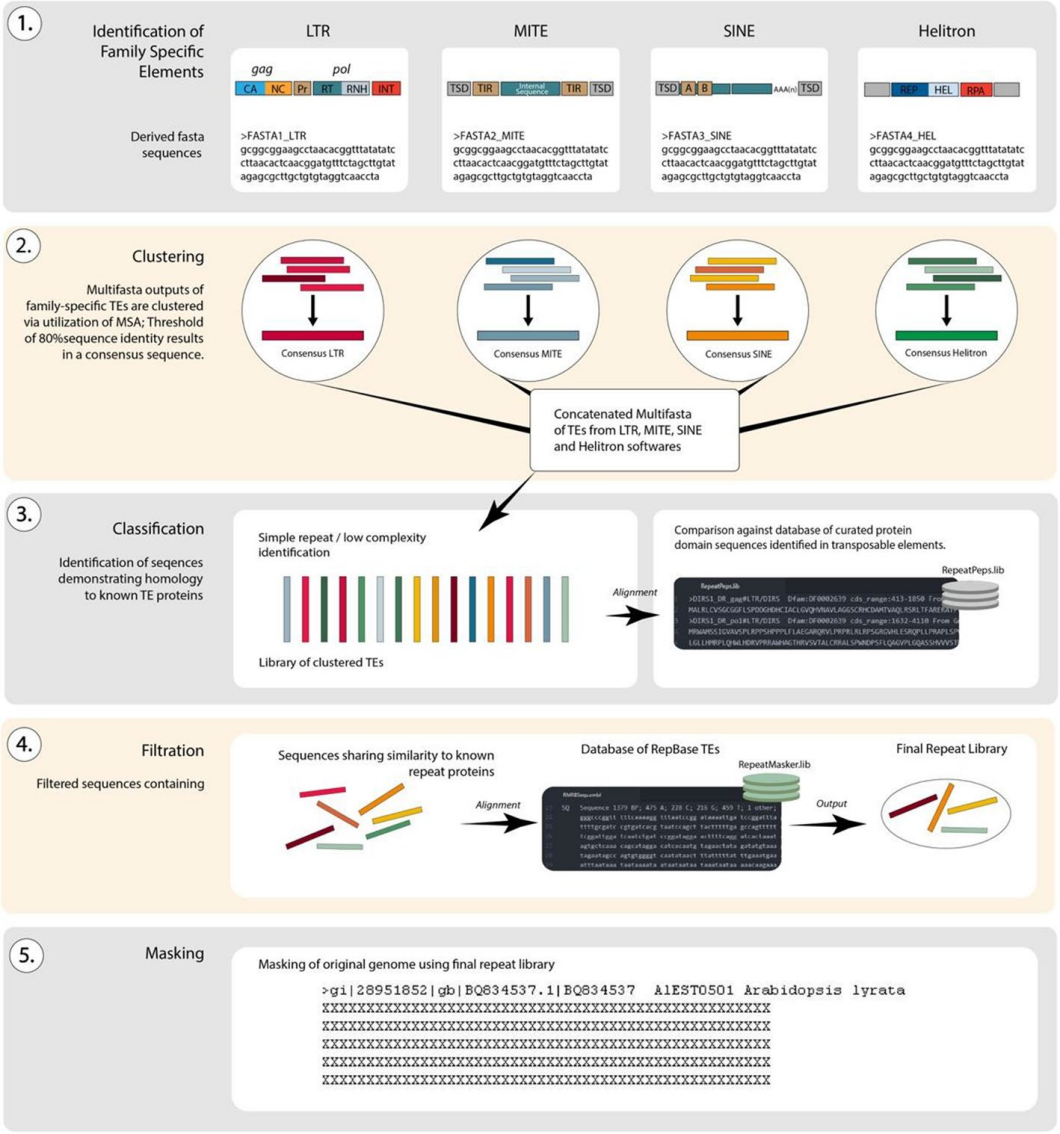
Overview of the RepBox pipeline. Genome sequences in standard fasta format are used as input in different family-specific detection tools. The output of each tool is then clustered, classified, and filtered using custom pipeline scripts. The final repeat library is then used to create a masked genome with repeat sequences blocked out for downstream analyses

RepBox incorporates existing TE detection software and uses custom scripts to process, filter, and aggregate the output of each separate tool (Fig. 1). In the first step, RepBox uses the following repeat identification packages; RepeatModeler (version 2.0.1) [10]; HelitronScanner [11], SINE_Scan [12], and MITEFinderII [13]. Our pipeline consists of three-phases: (1) Baseline repeat annotation of the genome using RepeatModeler, (2) Identification of TE superfamilies using de novo software and (3) consolidation of repeat families and final masking of the original genome. Genomes used for benchmarking were *Arabidopsis thaliana* (TAIR10, INSDC Assembly GCA_000001735.1) and *Oryza sativa* (IRGSP-1.0, INSDC Assembly GCA_001433935.1). Both genomes were retrieved from Ensembl [14] and selected due to their extensive use as model organisms and thoroughly curated annotations.

### Generation of a baseline repeat annotation

To compare the performance of RepBox with the existing TE detection program RepeatModeler, we used curated TE databases for 2 well-annotated plant genomes: *Arabidposis thaliana* (TAIR10) [15] and *Oryza sativa* (IRGSP Build 5) [16]. Percent composition of Class I (LTRs, non-LTRs) and Class II (MITEs, Helitrons) elements for both *A. thaliana* and *O. sativa* varies widely when characterizing each species. For instance, nearly 48% of the *O. sativa* genome is composed of TEs, with LTRs being the primary contributor. Conversely, *A. thaliana* contains a lower percentage of repetitive elements, ~ 14% of its genome, with Rolling-Circle/Helitron (RC/HELITRON) elements being the primary family of identified elements. *O. sativa* and other cultivated species of Poaceae, such as *Triticum aestivum* and *Zea mays*, are known to contain large quantities of TEs [2], even compared to other plant species. TE composition and profile are important to note, as both the abundance and diversity of TEs present within a given genome are major contributors to the computational complexity of identification.

For each genome, the reference transposon annotations were retrieved and parsed in R [17] with several third-party packages: dplyr (version 1.0.0) [18], chromPlot (version 1.16.0) [19], stringr (version 1.4.0) [20] and reticulate (version 1.16) [21]. Commands used in R for processing the reference GFF files are provided in Additional file 1.

### Identification of TE superfamilies: MITEs, Helitrons and SINEs

Identification of DNA transposons was performed by MITEFinder. Comparisons of two MITE identification tools, MITEFinder (version 0.1) [13] and MITETracker (version 1.0) [22], found that MITEFinder consistently identified a higher number of elements in both *Arabidopsis* and *Oryza* than MITETracker (Additional file 2: Tables S2 and S3). Thus, we selected MITEFinder as the optimal tool to integrate into our pipeline. Similarly, we also compared two different Helitron detection programs, and found that for our reference genomes HelitronScanner (version 1.0) [11] uncovered more Helitron candidates than the alternative program EAHelitron [23] (Additional file 2: Tables S4 and S5) and was the tool that we selected. Finally, for the identification of SINE elements, we used SINE_Scan (version 1.1.1) [12]. Each package used in the identification of family-specific elements provides additional resolution of the sequences reported as “unknowns” by RepeatModeler, TE detection software. Detailed commands for executing each software package are outlined in Additional file 1.

### Consensus repeat library clustering, filtration and genome masking

All fasta output files generated by the TE detection tools were clustered using VSEARCH (version 2.14 ) [24] to remove redundant sequences using an 80% sequence similarity criteria. Clustering of candidate sequences is a necessary process serving two purposes for the RepBox pipeline; (1) Sequences that are initially uncharacterized are potentially related to sequences with known homology but are too divergent to be identified by default in each software package, so finding similar sequence clusters increases the chances of their correct classification and (2) With multiple sources for characterization, there is a potential for redundancy in sequences that were identified independently in each package and this requires clustering to remove overlapping calls. Following clustering, filtration of false positives and protein-coding sequences corresponding to structural components previously observed in TEs is performed using the protocol outlined in Coghlan et al. [25]. Briefly, sequences close in homology to either known TEs or known protein domains are aligned using BLAST, and sequences with low percent identity compared to known TE proteins and domains are subsequently filtered out.

### Comparison of feature identification by different tools

After running RepeatModeler and RepBox on both reference genomes, we used bedtools (version 2.3) [26] to determine how consistently the positions and definitions of elements identified by each pipeline overlapped with the reference repeat annotations. For each class of element, we calculated the percentage of known reference elements that were correctly captured by each software package, and used these metrics to assess how well RepBox performed relative to RepeatModeler. False positives are calculated by subtracting the coutn of elements identified using a given method from the number of elements of a given class observed in the reference. True negatives are calculated by subtracting the count of false positive elements identified when calculating the false positives from the count of elements in a given class observed in the reference. The false positive rate is the proportion of falsely identified positive instances relative to all the instances classified as positive. Potentially novel elements were quantified by calculating the difference of true positives in each method of identification (RepeatMasker and RepBox). We first calculated true positives by utilizing the false positive rate multiplied by the number of positives. Following this, we calculated the potentially novel elements as the difference between RepBox true positives and RepeatMasker true positives.

## Results

### Comparisons of RepBox and RepeatModeler/RepeatMasker

**Fig. 2:**
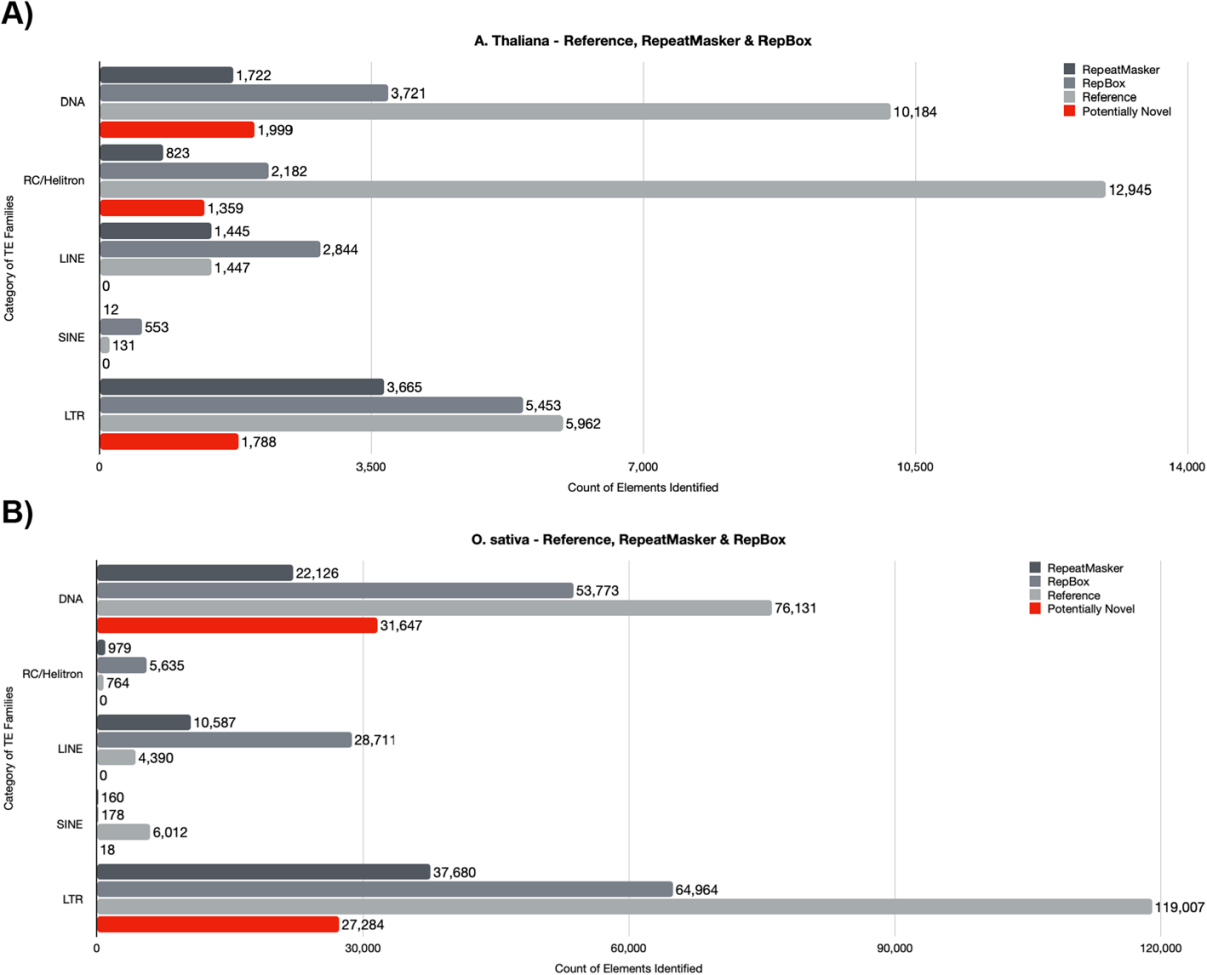
Comparison of results from RepeatModeler and RepBox.** A** The number of each class of element identified in *Arabidopsis thaliana*. Dark gray bars indicate the number of elements identified by RepeatMasker, mid-gray bars indicate the number of elements identified by RepBox, light gray bars indicate the number of known elements in the reference dataset and red bars indicate potentially novel; The actual number of each element is indicated in the text within each bar. **B** The same as panel (a), but with the results from the *Oryza sativa* genome

While neither RepBox nor RepeatModeler could re-capitulate the manually curated reference element counts for either genome that we examined, we did find that RepBox showed notable increases in the numbers of every class of element it identified when compared to RepeatModeler (Fig. 2A, B). In particular, RepBox classified 4% more DNA transposons than RepeatModeler in *A. thaliana* and 3% more in *O. sativa*. Of this, a significant proportion of that can be identified as potentially novel elements identified by RepBox (Fig. 2A, B, red bars). Increases in the number of other types of identified elements, including rRNA, satellite, simple repeat, sRNA, tRNA, were also observed in both organisms when comparing the RepBox analysis to RepeatModeler (Additional file 2: Tables S7, S7-B, S8 and S8-B). A total of 1445 LINE elements were identified by RepeatModeler in *A. thaliana*, while RepBox increased this count to 2844, nearly double the number identified by RepeatModelerSimilarly, RepBox called more than twice as many LINE elements in *O. sativa* (Fig. 2B). It is worth noting that LINE elements represent the only category of TE where RepBox identified more elements than were present in the initial reference annotation. This is likely the result of the structural characteristics of LINEs themselves (see Discussion).

SINE element candidate counts were also increased by a total of 551 in the RepBox analysis of *A. thaliana*, and were modestly increased by a total of 18 elements in *O. sativa*. Finally, the count of LTR elements was also higher in the RepBox analysis of both genomes; however, the total count of these elements still fell short of the overall number maintained in the reference databases, especially in the analysis of *O. sativa*. In spite of this, RepBox identified 1788 potentially novel LTRs in *A. thaliana* and 27,284 potentially novel LTRs identified in *O. sativa.* This highlights the fact that even though incorporating family-specific detection software certainly improves RepBox’s ability to identify a higher number and diversity of elements, there is not yet any automated method that can match manual curation of TEs [27].

## Discussion

In our analysis we observed that RepBox effectively identified a greater number of elements and a higher diversity of TE families than running RepeatModeler alone. While in most categories of elements RepBox still fell short of the reference counts identified through automated and manual curation, our results do indicate that integrating multiple existing structure and homology-based detection tools in a single pipeline significantly improves the characterization of the TE landscape in a given genome. Interestingly, LINE elements were the only category of elements where RepBox actually appeared to overcall the number of sequences.

We believe this is likely due to the structure of LINE elements themselves, as well as the current lack of any de novo software designed for the specific detection of LINEs. Most observed elements are predominately of the L1 superfamily found in mammalian genomes [28], and detection options beyond insertion site polymorphisms are sparse, making LINEs the only superfamily of transposable elements lacking dedicated software that can utilize structural characteristics for detection. Structurally, LINE elements are naturally more dispersed than other element types, spanning thousands of base pairs and typically containing multiple coding regions. Programs like HelitronScanner and MITEFinder, which are implemented as part of RepBox, may actually recognize *fragments* of LINE elements as being structurally indicative of TEs, but they cannot accurately identify these fragments as belonging to the same large element. Subsequently, when these fragments are clustered with known database elements as part of RepeatModeler, each fragment is found to be closely homologous with a LINE element, but again they are not correctly assigned as a single LINE element, which falsely inflates the number of LINE elements called by RepBox. We feel that the development of methods aimed at the detection of LINEs and the improvement of current TE databases will be necessary to increase the accuracy of automated TE detection software.

## Conclusions

With the onset of next-generation sequencing, copious quantities of genomic data has led to a surplus of software available for the analysis of that data, and in particular for the identification of transposable elements. Here we describe our pipeline RepBox, a novel analysis pipeline that utilizes newer software and builds upon prior annotation processes by incorporating family-specific identification methods with more traditional repeat detection programs. We demonstrate that our pipeline shows significant increases in the calling of DNA, non-LTR, and Helitron/RC elements in two different plant genomes when compared with standard TE annotation software. We also provide our pipeline in a freely available, easy to install suite of scripts that can be downloaded from GitHub at: https://github.com/shelvasha/RepBox.

### Availability and requirements

**Project name**: RepBox

**Project home page**: e.g. https://github.com/shelvasha/RepBox

**Operating system(s)**: UNIX, Linux

**Programming language**: Bash, Python

**Other requirements**: Python 3.0 or higher

**License**: e.g. GNU GPL, FreeBSD etc.

Any restrictions to use by non-academics: e.g. license needed

### Supplementary information

**Additional file 1**: Code.

**Additional file 2**: Tables.

## Data Availability

All code for installing and running the RepBox pipeline is available at https://github.com/shelvasha/RepBox.

## Abbreviations

LINE: Long Interspersed Nuclear Elements
LTR: Long Terminal Repeat
MITE: Miniature Inverted Terminal Elements
SINE: Short Interspersed Nuclear Elements
TE: Transposable Elements
