# Supplementary material for: RepBox: a toolbox for the identification of repetitive elements
Source: BMC Bioinformatics. 2023 Aug 22;24:317. doi: 10.1186/s12859-023-05419-5 (PMC10463291; doi:10.1186/s12859-023-05419-5)
Supplement: Supplementary file 1 — Additional file 1: Code. [file 12859_2023_5419_MOESM1_ESM.docx]

**Supplementary File 1, Code**

# Comparison of Reference GFFs of *O. sativa* and *A. thaliana*

## R, Bash & Python Commands

### Library Initiation

library("dplyr")

library("chromPlot")

library("stringr")

library("reticulate")

library("rtracklayer")

### Reading in Reference GFF

Oryza_ref <- read.delim("~/R\ Scripts/irgsp1_repeat_unit.gff",header=FALSE)

names(Oryza_ref)[1:9] <- c("Chrom","Source","Type","Start","End","Score","Strand","Phase","Attributes")

Oryza_ref$Chrom <- str_replace(Oryza_ref$Chrom, "chr0","")

Oryza_ref$Chrom <- str_replace(Oryza_ref$Chrom, "chr","")

export(Oryza_ref,con = "Oryza_reference.gff3", format="gff3")

# Helitron Comparisons

### Parsing Helitrons from Reference GFFs

helitron_reference <- Oryza_ref[str_detect(Oryza_ref$Attributes,"#RC"),]

helitron_reference_format <- dplyr::select(helitron_reference, c(1,4,5))

export(helitron_reference,con="Oryza_helitron_reference.gff3", format="gff3")

output1 <- data.frame(str_remove(str_split(str_split(toString(helitron_reference[9][1]),"Name=", simplify = TRUE),",", simplify = TRUE)[,1],'"'))

output1 <- data.frame(output1[2:nrow(output1),])

output1[nrow(output1),]<- str_sub(output1[nrow(output1),], end=-2)

write.table(output1, file="Oryza_HEL.txt", quote=FALSE, row.names=FALSE, col.names=FALSE)

### EAHelitron Compared to Reference (*O. sativa*)

# Bash commands

bedtools intersect -a ~/R\ Scripts/Oryza_reference.gff3 -b ~/results/results_Oryza_sativa/eahelitron_out/EAHeli_out.gff3 -u -f 0.8 > Oryza_ref_Helitron_EAHelitron_overlap.gff3

### HelitronScanner Compared to Reference

# Bash commands

bedtools intersect -a ~/R\ Scripts/Oryza_reference.gff3 -b ~/results/results_Oryza_sativa/helitronscanner_out/helitronscanner_out.Oryza_sativa.hel.gff3 -u -f 0.8 > Oryza_ref_Helitron_HelitronScanner_overlap.gff3

### Helitron Counts & Comparisons of Software

Oryza_ea_helitron_count <- nrow(Oryza_ea_helitron_gff)

Oryza_helitron_scanner_count <- nrow(Oryza_helitron_scanner_gff)

Oryza_ref_helitron_count <- nrow(helitron_reference)

barplot(cbind(Oryza_ref_helitron_count,Oryza_ea_helitron_count, Oryza_helitron_scanner_count))

helitron_table <- data.frame(rbind(Oryza_ref_helitron_count,Oryza_ea_helitron_count, Oryza_helitron_scanner_count))

bedtools intersect -a ~/results/results_Oryza_sativa/eahelitron_out/EAHeli_out.gff3 -b ~/results/results_Oryza_sativa/helitronscanner_out/helitronscanner_out.Oryza_sativa.hel.gff3 -wao > Oryza_EA_HS_overlap.gff3

Oryza_EA_HS <- read.delim("Oryza_EA_HS_overlap.gff3")

names(Oryza_EA_HS)[1:9] <- c("Chrom","Source","Type","Start","End","Score","Strand","Phase","Attributes")

Oryza_EA_HS

write.table(Oryza_EA_HS, file="Oryza_EAHelitron-Helitronscanner_comparision.txt", quote=FALSE, row.names=FALSE, col.names=FALSE)

# MITE Comparisons

### Parsing MITEs from Reference GFFs (*O. sativa*)

Oryza_mite_reference <- Oryza_ref[str_detect(Oryza_ref$Attributes,"#DNA/Mite"),]

export(Oryza_mite_reference,con="Oryza_mite_reference.gff3", format="gff3")

output2 <- data.frame(str_remove(str_split(str_split(toString(Oryza_mite_reference[9][1]),"Name=", simplify = TRUE),",", simplify = TRUE)[,1],'"'))

output2 <- data.frame(output2[2:nrow(output2),])

output2[nrow(output2),]<- str_sub(output2[nrow(output2),], end=-2)

write.table(output2, file="Oryza_DNA.txt", quote=FALSE, row.names=FALSE, col.names=FALSE)

Oryza_dna_profile <- read.delim("Oryza_DNA_OUT.txt",header=FALSE)

Oryza_dna_profile <- data.frame(table(Oryza_dna_profile))

names(Oryza_dna_profile) <- c("Transposon Family","Count")

##

###

### MITETracker Comparison to Reference

# Bash commands:

bedtools intersect -a ~/R\ Scripts/Oryza_reference.gff3 -b ~/results/results_Oryza_sativa/mitetracker_out/Oryza_sativa/all.gff3 -r -u -f 0.8 > Oryza_ref_MITE_MITETracker_overlap.gff3

### MITEFinder Comparison to Reference

# Bash commands

bedtools intersect -a ~/R\ Scripts/Oryza_reference.gff3 -b ~/results/results_Oryza_sativa/mitefinder_out/mitefinder.gff3 -u -f 0.8 > Oryza_ref_MITE_MITEFinder_overlap.gff3

### MITE Counts

Oryza_mitefinder_count <- nrow(Oryza_mitefinder)

Oryza_mitetracker_count <- nrow(Oryza_mitetracker)

Oryza_mite_reference_count <- nrow(Oryza_mite_reference)

barplot(cbind(Oryza_mite_reference_count, Oryza_mitefinder_count, Oryza_mitetracker_count))

bedtools intersect -a ~/results/results_Oryza_sativa/mitetracker_out/Oryza_sativa/all.gff3 -b ~/results/results_Oryza_sativa/mitefinder_out/mitefinder.gff3 -r -f 0.8 > Oryza_MT_MF_overlap.gff3

Oryza_MT_MF <- read.delim("Oryza_MT_MF_overlap.gff3")

names(Oryza_MT_MF)[1:9] <- c("Chrom","Source","Type","Start","End","Score","Strand","Phase","Attributes")

write.table(Oryza_MT_MF, file="Oryza_MITEFinder-MITETracker_comparision.txt", quote=FALSE, row.names=FALSE, col.names=FALSE)

# SINEs

### Reading in Reference

Oryza_sine_reference <- Oryza_ref[str_detect(Oryza_ref$Attributes,"#SINE"),]

export(Oryza_sine_reference,con="Oryza_sine_reference.gff3", format="gff3")

output3 <- data.frame(str_remove(str_split(str_split(toString(Oryza_sine_reference[9][1]),"Name=", simplify = TRUE),",", simplify = TRUE)[,1],'"'))

output3 <- data.frame(output3[2:nrow(output3),])

output3[nrow(output3),]<- str_sub(output3[nrow(output3),], end=-2)

write.table(output3, file="Oryza_SINE.txt", quote=FALSE, row.names=FALSE, col.names=FALSE)

Oryza_sine_profile <- read.delim("Oryza_SINE.txt",header=FALSE)

Oryza_sine_profile <- data.frame(table(Oryza_sine_profile))

names(Oryza_sine_profile) <- c("Transposon Family","Count")

Oryza_sine_profile

write.table(Oryza_sine_profile, file="Oryza_SINE_TE_profile.txt", quote=FALSE, row.names=FALSE, sep=",")

### GFF Comparisons SineScan to Reference

bedtools intersect -a ~/results/results_Oryza_sativa/comparison/Oryza_MITE_reference_annotation.gff -b /Users/shelvasha/Repbox/mitetracker_out/Oryza_sativa/all.gff3 > ref_MITE_MITETracker_overlap.gff3

###

### SINEScan Counts

Oryza_ref_sinescan_compare <- read.delim("",header=FALSE)

names(Oryza_ref_sinescan_compare)[1:9] <- c("Chrom","Source","Type","Start","End","Score","Strand","Phase","Attributes")

Oryza_ref_sinescan_compare_format <- dplyr::select(Oryza_ref_sinescan_compare, c(1,4,5))

Splot <- chromPlot(annot1 = Oryza_ref_sinescan_compare_format)

SINE Counts

Oryza_sinescan_count <- nrow(Oryza_sinescan)

Oryza_sine_reference_count <- nrow(Oryza_sine_reference)

### 
