# Supplementary material for: RepBox: a toolbox for the identification of repetitive elements
Source: BMC Bioinformatics. 2023 Aug 22;24:317. doi: 10.1186/s12859-023-05419-5 (PMC10463291; doi:10.1186/s12859-023-05419-5)
Supplement: Supplementary file 2 — Additional file 2: Tables. [file 12859_2023_5419_MOESM2_ESM.docx]

**Supplementary File 2, Tables**

Table 1, Parameterization of HelitronScanner

| **Fuzziness Setting** | **CTRRt 3' Terminal** | **Identified Elements** |
| --- | --- | --- |
| 0 | CTAGT | 665 |
| 1 | CT[AG]GT | 732 |
| 2 | CTA[AG]T | 782 |
| 3 | CT[AG]{2}T | 950 |
| 4 | CT[AG]{2}.{1} | 1808 |
| 5 | CTAG.{1} | 828 |

Table 2, MITE Analysis of *O. sativa*

| **Software** | **Total MITE Count** | **Counts of overlaps w/Reference** | **Overlaps >= 80% Sequence Identity** | **% of Sequences Overlap with Ref.** | **Ref.** | **Average overlap length (bps)** | **FP** | **TN** | **FPR (%)** |
| --- | --- | --- | --- | --- | --- | --- | --- | --- | --- |
| **MITETracker** | 17,700 | 19,018 | 17,094 | 89.88% | 76131 | 249.9713 | 1924 | 17094 | 10.12% |
| **MITEFinder** | 40,814 | 24,028 | 5,918 | 24.63% |  | 203.5359 | 18110 | 5918 | 75.37% |

Summary table of MITE analysis of *O.sativa*. Abbreviations: **Ref**. (count of reference MITE elements), **FP** (count of false positive), **TN** (count of calculated true negatives), **FPR** (Calculated false positive rate).

Table 3, MITE Analysis of *A. thaliana*

| **Software** | **Total MITE Count** | **Counts of overlaps w/Reference** | **Overlaps >= 80% Sequence Identity** | **% of Sequences Overlap with Ref.** | **Ref.** | **Average overlap length (bps)** | **FP** | **TN** | **FPR (%)** |
| --- | --- | --- | --- | --- | --- | --- | --- | --- | --- |
| **MITETracker** | 230 | 265 | 126 | 47.55% | 10184 | 382.8889 | 139 | 126 | 52.45% |
| **MITEFinder** | 18,576 | 9,796 | 2,032 | 20.74% |  | 253.9972 | 7764 | 2032 | 79.26% |

Summary table of MITE analysis of *A.thaliana*. Abbreviations: **Ref**.(count of reference MITE elements), **FP** (count of false positive), **TN** (count of calculated true negatives), **FPR** (Calculated false positive rate).

Table 4, Helitron Analysis of *O. sativa*

| **Software** | **Total Helitron Count** | **Counts of overlaps w/Reference** | **Overlaps >= 80% Sequence Identity** | **% of Sequences Overlap with Ref.** | **Ref.** | **Average overlap length (bps)** | **FP** | **TN** | **FPR (%)** |
| --- | --- | --- | --- | --- | --- | --- | --- | --- | --- |
| **EAHelitron** | 3,316 | 2,022 | 29 | 1.43% | 764 | 24.93719 | 1993 | 29 | 98.57% |
| **HelitronScanner** | 3,447 | 23,922 | 22,252 | 93.02% |  | 469.1358 | 1670 | 22252 | 6.98% |

Summary table of Helitron analysis of *O.sativa*. Abbreviations: **Ref**.(count of reference helitron elements), **FP** (count of false positive), **TN** (count of calculated true negatives), **FPR** (Calculated false positive rate).

Table 5, Helitron Analysis of *A. thaliana*

| **Software** | **Total Helitron Count** | **Counts of overlaps w/Reference** | **Overlaps >= 80% Sequence Identity** | **% of Sequences Overlap with Ref.** | **Ref.** | **Average overlap length (bps)** | **FP** | **TN** | **FPR (%)** |
| --- | --- | --- | --- | --- | --- | --- | --- | --- | --- |
| **EAHelitron** | 665 | 604 | 11 | 4.67% | 12945 | 26.4404 | 593 | 11 | 98.18% |
| **HelitronScanner** | 441 | 1,842 | 1,536 | 14.23% |  | 731.9224 | 306 | 1536 | 16.61% |

Summary table of Helitron analysis of *A.thaliana*. Abbreviations: **Ref**.(count of reference helitron elements), **FP** (count of false positive), **TN** (count of calculated true negatives), **FPR** (Calculated false positive rate).

Table 7, RepeatModeler/Masker & Repbox Analysis of *A. thaliana*

| RepeatMasker | Family | Count | Total | Repbox | Family | Count | Total | Reference Total |
| --- | --- | --- | --- | --- | --- | --- | --- | --- |
| DNA | DNA | 5 | 1722 | DNA | DNA | 19 | 3721 | 10184 |
|  | DNA/CMC-Chapaev | 2 |  |  | DNA/CMC-Chapaev | 0 |  |  |
|  | DNA/CMC-EnSpm | 540 |  |  | DNA/CMC-EnSpm | 1323 |  |  |
|  | DNA/CMC-Transib | 0 |  |  | DNA/CMC-Transib | 15 |  |  |
|  | DNA/Dada | 5 |  |  | DNA/Dada | 9 |  |  |
|  | DNA/hAT | 1 |  |  | DNA/hAT | 0 |  |  |
|  | DNA/hAT-Ac | 172 |  |  | DNA/hAT-Ac | 113 |  |  |
|  | DNA/hAT-Charlie | 1 |  |  | DNA/hAT-Charlie | 10 |  |  |
|  | DNA/hAT-Tag1 | 0 |  |  | DNA/hAT-Tag1 | 8 |  |  |
|  | DNA/hAT-Tip100 | 97 |  |  | DNA/hAT-Tip100 | 169 |  |  |
|  | DNA/IS3EU | 0 |  |  | DNA/IS3EU | 18 |  |  |
|  | DNA/Kolobok-T2 | 5 |  |  | DNA/Kolobok-T2 | 0 |  |  |
|  | DNA/Merlin | 0 |  |  | DNA/Merlin | 3 |  |  |
|  | DNA/MULE-MuDR | 782 |  |  | DNA/MULE-MuDR | 1454 |  |  |
|  | DNA/P | 0 |  |  | DNA/P | 0 |  |  |
|  | DNA/PiggyBac | 1 |  |  | DNA/PiggyBac | 2 |  |  |
|  | DNA/PIF-Harbinger | 97 |  |  | DNA/PIF-Harbinger | 480 |  |  |
|  | DNA/TcMar-ISRm11 | 0 |  |  | DNA/TcMar-ISRm11 | 0 |  |  |
|  | DNA/TcMar-Pogo | 12 |  |  | DNA/TcMar-Pogo | 87 |  |  |
|  | DNA/TcMar-Stowaway | 0 |  |  | DNA/TcMar-Stowaway | 9 |  |  |
|  | DNA/TcMar-Tc1 | 0 |  |  | DNA/TcMar-Tc1 | 0 |  |  |
|  | DNA/TcMar-Tc2 | 1 |  |  | DNA/TcMar-Tc2 | 0 |  |  |
|  | DNA/Zisupton | 1 |  |  | DNA/Zisupton | 2 |  |  |
| RC/Helitron | RC/Helitron | 806 | 823 | RC/Helitron | RC/Helitron | 2182 | 2182 | 12945 |
|  | Helitron-2 | 17 |  |  | Helitron-2 | 0 |  |  |
| LINE | LINE/I | 2 | 1445 | LINE | LINE/I | 0 | 2844 | 1447 |
|  | LINE/I-Jockey | 1 |  |  | LINE/I-Jockey | 0 |  |  |
|  | LINE/L1 | 1429 |  |  | LINE/L1 | 2842 |  |  |
|  | LINE/L1-Tx1 | 5 |  |  | LINE/L1-Tx1 | 1 |  |  |
|  | LINE/L2 | 2 |  |  | LINE/L2 | 0 |  |  |
|  | LINE/Penelope | 4 |  |  | LINE/Penelope | 1 |  |  |
|  | LINE/R1 | 1 |  |  | LINE/R1 | 0 |  |  |
|  | LINE/RTE-X | 1 |  |  | LINE/RTE-X | 0 |  |  |
| SINE | SINE | 2 | 12 | SINE | SINE | 38 | 553 | 131 |
|  | SINE/5S-Deu-L2 | 0 |  |  | SINE/5S-Deu-L2 | 501 |  |  |
|  | SINE/ID | 10 |  |  | SINE/ID | 14 |  |  |
| LTR | LTR | 5 | 3665 | LTR | LTR | 3 | 5453 | 5962 |
|  | LTR/Caulimovirus | 0 |  |  | LTR/Caulimovirus | 2 |  |  |
|  | LTR/Copia | 947 |  |  | LTR/Copia | 1403 |  |  |
|  | LTR/ERV1 | 8 |  |  | LTR/ERV1 | 13 |  |  |
|  | LTR/ERVK | 13 |  |  | LTR/ERVK | 107 |  |  |
|  | LTR/Gypsy | 2666 |  |  | LTR/Gypsy | 3892 |  |  |
|  | LTR/Ngaro | 13 |  |  | LTR/Ngaro | 0 |  |  |
|  | LTR/Pao | 13 |  |  | LTR/Pao | 33 |  |  |
| Other | rRNA | 13 | 65483 | Other | rRNA | 102 | 94564 | NA |
|  | Satellite | 19 |  |  | Satellite | 113 |  |  |
|  | Simple_repeat | 0 |  |  | Simple_repeat | 29832 |  |  |
|  | snRNA | 14 |  |  | snRNA | 48 |  |  |
|  | tRNA | 84 |  |  | tRNA | 542 |  |  |
|  | Unknown | 65353 |  |  | Unknown | 63927 |  |  |
|  | Total | 68234 |  |  | Total | 117561 |  |  |

Table 7-B, RepeatModeler/Masker & Repbox Analysis of *A. thaliana* (simplified)

| **Family** | **RM** | **RB** | **Ref.** | **RM FP** | **RM TN** | **RM FPR** | **RB FP** | **RB TN** | **RB FPR** | **Novel** |
| --- | --- | --- | --- | --- | --- | --- | --- | --- | --- | --- |
| **DNA** | 1722 | 3721 | 10184 | 0 | 10184 | 0.00% | 0 | 10184 | 0.00% | 1999 |
| **RC/Helitron** | 823 | 2182 | 12945 | 0 | 12945 | 0.00% | 0 | 12945 | 0.00% | 1359 |
| **LINE** | 1445 | 2844 | 1447 | 0 | 1447 | 0.00% | 1397 | 50 | 96.54% | 0 |
| **SINE** | 12 | 553 | 131 | 0 | 131 | 0.00% | 422 | 0 | 100.00% | 0 |
| **LTR** | 3665 | 5453 | 5962 | 0 | 5962 | 0.00% | 0 | 5962 | 0.00% | 1788 |

Simplified summary table of RepeatMasker analysis of A*.thaliana*. Abbreviations: **RM** (counts of elements identified in Repeatmasker), **RB** (counts of elements identified in RepBox), **Ref**.(counts of reference elements), **RM FP** (count of false positive for RepeatMasker), **RM** **TN** (count of calculated true negatives for RepeatMasker), **RM** **FPR** (Calculated false positive rate for RepeatMasker). **RB FP** (count of false positive for RepBox), **RB** **TN** (count of calculated true negatives for RepBox), **RB** **FPR** (Calculated false positive rate for RepBox), **Novel** (Count of potential novel elements based on false positive rates for RepeatMasker and RepBox).

Table 8, RepeatModeler/Masker & Repbox Analysis of *Oryza sativa*

| RepeatMasker | Family | Count | Total | Repbox | Family | Count | Total | Reference Total |
| --- | --- | --- | --- | --- | --- | --- | --- | --- |
| DNA | DNA/CMC-EnSpm | 9765 | 22126 | DNA | DNA/CMC-EnSpm | 26020 | 53773 | 76131 |
|  | DNA/DNA | 2306 |  |  | DNA/DNA | 2844 |  |  |
|  | DNA/Ginger-1 | 0 |  |  | DNA/Ginger-1 | 203 |  |  |
|  | DNA/hAT | 0 |  |  | DNA/hAT | 226 |  |  |
|  | DNA/hAT-Ac | 2186 |  |  | DNA/hAT-Ac | 7262 |  |  |
|  | DNA/hAT-Charlie | 0 |  |  | DNA/hAT-Charlie | 720 |  |  |
|  | DNA/hAT-Tag1 | 243 |  |  | DNA/hAT-Tag1 | 490 |  |  |
|  | DNA/hAT-Tip100 | 1296 |  |  | DNA/hAT-Tip100 | 4461 |  |  |
|  | DNA/IS3EU | 814 |  |  | DNA/IS3EU | 358 |  |  |
|  | DNA/Kolobok-T2 | 133 |  |  | DNA/Kolobok-T2 | 1030 |  |  |
|  | DNA/Kolobok-H | 0 |  |  | DNA/Kolobok-H | 131 |  |  |
|  | DNA/Maverick | 0 |  |  | DNA/Maverick | 173 |  |  |
|  | DNA/Merlin | 821 |  |  | DNA/Merlin | 161 |  |  |
|  | DNA/MULE-MuDR | 3546 |  |  | DNA/MULE-MuDR | 5412 |  |  |
|  | DNA/P | 0 |  |  | DNA/P | 292 |  |  |
|  | DNA/PIF-Harbinger | 741 |  |  | DNA/PIF-Harbinger | 2337 |  |  |
|  | DNA/TcMar | 0 |  |  | DNA/TcMar | 157 |  |  |
|  | DNA/TcMar-ISRm11 | 0 |  |  | DNA/TcMar-ISRm11 | 132 |  |  |
|  | DNA/TcMar-Stowaway | 275 |  |  | DNA/TcMar-Stowaway | 1364 |  |  |
| RC/Helitron | RC/Helitron | 979 | 979 | RC/Helitron | RC/Helitron | 4975 | 5635 | 764 |
|  | Helitron-2 | 0 |  |  | Helitron-2 | 660 |  |  |
| LINE | LINE/I-Jockey | 393 | 10587 | LINE | LINE/I-Jockey | 0 | 28711 | 4390 |
|  | LINE/L1 | 10194 |  |  | LINE/L1 | 26389 |  |  |
|  | LINE/L1-Tx1 | 0 |  |  | LINE/L1-Tx1 | 1790 |  |  |
|  | LINE/L2 | 0 |  |  | LINE/L2 | 65 |  |  |
|  | LINE/Penelope | 0 |  |  | LINE/Penelope | 153 |  |  |
|  | LINE/RTE-BovB | 0 |  |  | LINE/RTE-BovB | 1 |  |  |
|  | LINE/RTE-X | 0 |  |  | LINE/Rex-Babar | 313 |  |  |
| SINE | SINE/ID | 0 | 160 | SINE | SINE/ID | 24 | 178 | 6012 |
|  | SINE/SINE | 160 |  |  | SINE/SINE | 154 |  |  |
| LTR | LTR/Caulimovirus | 252 | 37680 | LTR | LTR/Caulimovirus | 135 | 64964 | 119007 |
|  | LTR/Copia | 9401 |  |  | LTR/Copia | 17586 |  |  |
|  | LTR/ERV1 | 0 |  |  | LTR/ERV1 | 569 |  |  |
|  | LTR/ERVK | 1554 |  |  | LTR/ERVK | 1482 |  |  |
|  | LTR/ERVL | 0 |  |  | LTR/ERVL | 374 |  |  |
|  | LTR/Gypsy | 25939 |  |  | LTR/Gypsy | 39870 |  |  |
|  | LTR/LTR | 269 |  |  | LTR/LTR | 2171 |  |  |
|  | LTR/Ngaro | 265 |  |  | LTR/Ngaro | 1702 |  |  |
|  | LTR/Pao | 0 |  |  | LTR/Pao | 1075 |  |  |
| Other | Low_complexity | 9963 | 448316 | Other | Low_complexity | 8219 | 567021 | 77763 |
|  | rRNA | 697 |  |  | rRNA | 600 |  |  |
|  | Satellite | 54 |  |  | Satellite | 409 |  |  |
|  | Simple_repeat | 92147 |  |  | Simple_repeat | 80855 |  |  |
|  | snRNA | 54 |  |  | snRNA | 54 |  |  |
|  | tRNA | 318 |  |  | tRNA | 2475 |  |  |
|  | Unknown | 345083 |  |  | Unknown | 474409 |  |  |
|  | Total | 519888 | |  | Total | 721557 | |  |

Table 8-B, RepeatModeler/Masker & Repbox Analysis of *Oryza sativa* (Simplified)

| **Family** | **RM** | **RB** | **Ref.** | **RM FP** | **RM TN** | **RM FPR** | **RB FP** | **RB TN** | **RB FPR** | **Novel** |
| --- | --- | --- | --- | --- | --- | --- | --- | --- | --- | --- |
| **DNA** | 22126 | 53773 | 76131 | 0 | 76131 | 0.00% | 0 | 76131 | 0.00% | 31647 |
| **RC/Helitron** | 979 | 5635 | 764 | 215 | 549 | 28.14% | 4871 | 0 | 100.00% | 0 |
| **LINE** | 10587 | 28711 | 4390 | 6197 | 0 | 100.00% | 24321 | 0 | 100.00% | 0 |
| **SINE** | 160 | 178 | 6012 | 0 | 6012 | 0.00% | 0 | 6012 | 0.00% | 18 |
| **LTR** | 37680 | 64964 | 119007 | 0 | 119007 | 0.00% | 0 | 119007 | 0.00% | 27284 |

Simplified summary table of RepeatMasker analysis of O*. sativa*. Abbreviations: **RM** (counts of elements identified in Repeatmasker), **RB** (counts of elements identified in RepBox), **Ref**.(counts of reference elements), **RM FP** (count of false positive for RepeatMasker), **RM** **TN** (count of calculated true negatives for RepeatMasker), **RM** **FPR** (Calculated false positive rate for RepeatMasker). **RB FP** (count of false positive for RepBox), **RB** **TN** (count of calculated true negatives for RepBox), **RB** **FPR** (Calculated false positive rate for RepBox), **Novel** (Count of potential novel elements based on false positive rates for RepeatMasker and RepBox).
